# Author Correction: Fermionic order by disorder in a van der Waals antiferromagnet

**DOI:** 10.1038/s41598-023-29760-0

**Published:** 2023-02-20

**Authors:** R. Okuma, D. Ueta, S. Kuniyoshi, Y. Fujisawa, B. Smith, C. H. Hsu, Y. Inagaki, W. Si, T. Kawae, H. Lin, F. C. Chuang, T. Masuda, R. Kobayashi, Y. Okada

**Affiliations:** 1 Quantum Materials Science Unit, Okinawa Institute of Science and Technology (OIST), Onna, Okinawa 904‑0495 Japan; 2 Faculty of Science, University of the Ryukyus, Nishihara, Okinawa 903‑0213 Japan; 3 Department of Physics, National Sun Yat-Sen University, Kaohsiung, 80424 Taiwan; 4 Department of Applied Quantum Physics, Kyushu University, Fukuoka, 819‑0395 Japan; 5 Institute of Physics, Academia Sinica, Taipei, Taiwan; 6 The National Center for Theoretical Sciences, PhysicsDivision, Hsinchu, 30013 Taiwan; 7 Institute for Solid State Physics (ISSP), The University of Tokyo, Kashiwa, Chiba 277‑8581 Japan

Correction to: *Scientific Reports* 10.1038/s41598-020-72300-3, published online 17 September 2020

This Article contains errors in Figures 1, 2, 3 and 4, where the labels in the figures are missing. The correct Figures 1, 2, 3 and 4 and accompanying legends appear below.

**Figure 1:**
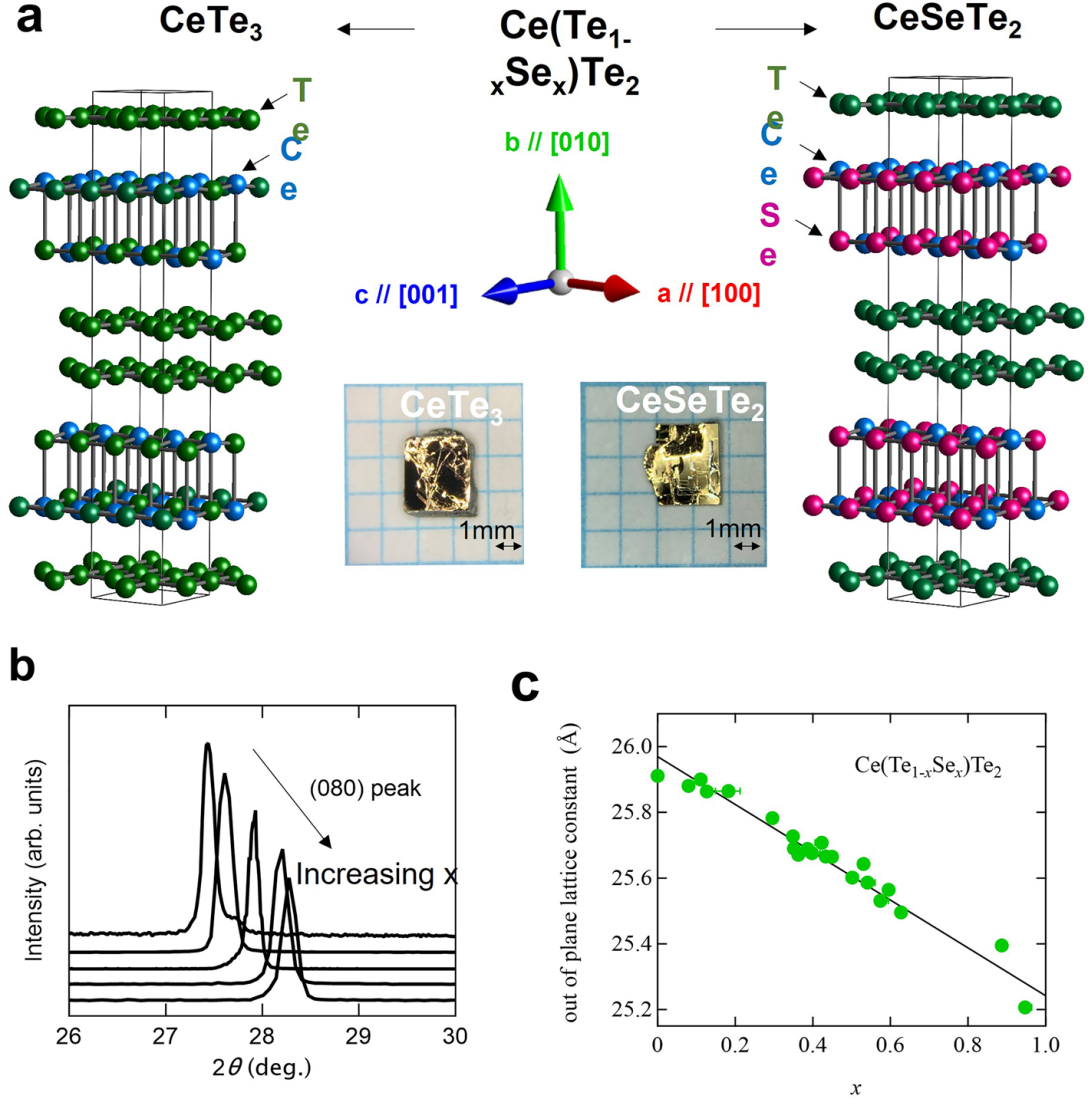
Systematic change of chemical pressure by iso-valent elemental substitution in a vdW coupled material Ce(Se_*x*_Te_1−*x*_)Te_2_. (**a**) Crystal structures of CeTe_3_ (left) and CeSeTe_2_ (right). Substituted Se atoms enter the magnetic blocking layer selectively. The typical picture of single crystals and the definition of crystallographic directions are also shown. (**b**) Doping dependence of characteristic X-ray diffraction (XRD) patterns near the (0 8 0) peak for Ce(Se_*x*_Te_1−*x*_)Te_2_. (**c**) The out of plane lattice constant *b* as a function of doping x determined from energy dispersive X-ray spectrometry (EDX). This relation was obtained by performing both XRD and EDX on individual crystal flakes.

**Figure 2:**
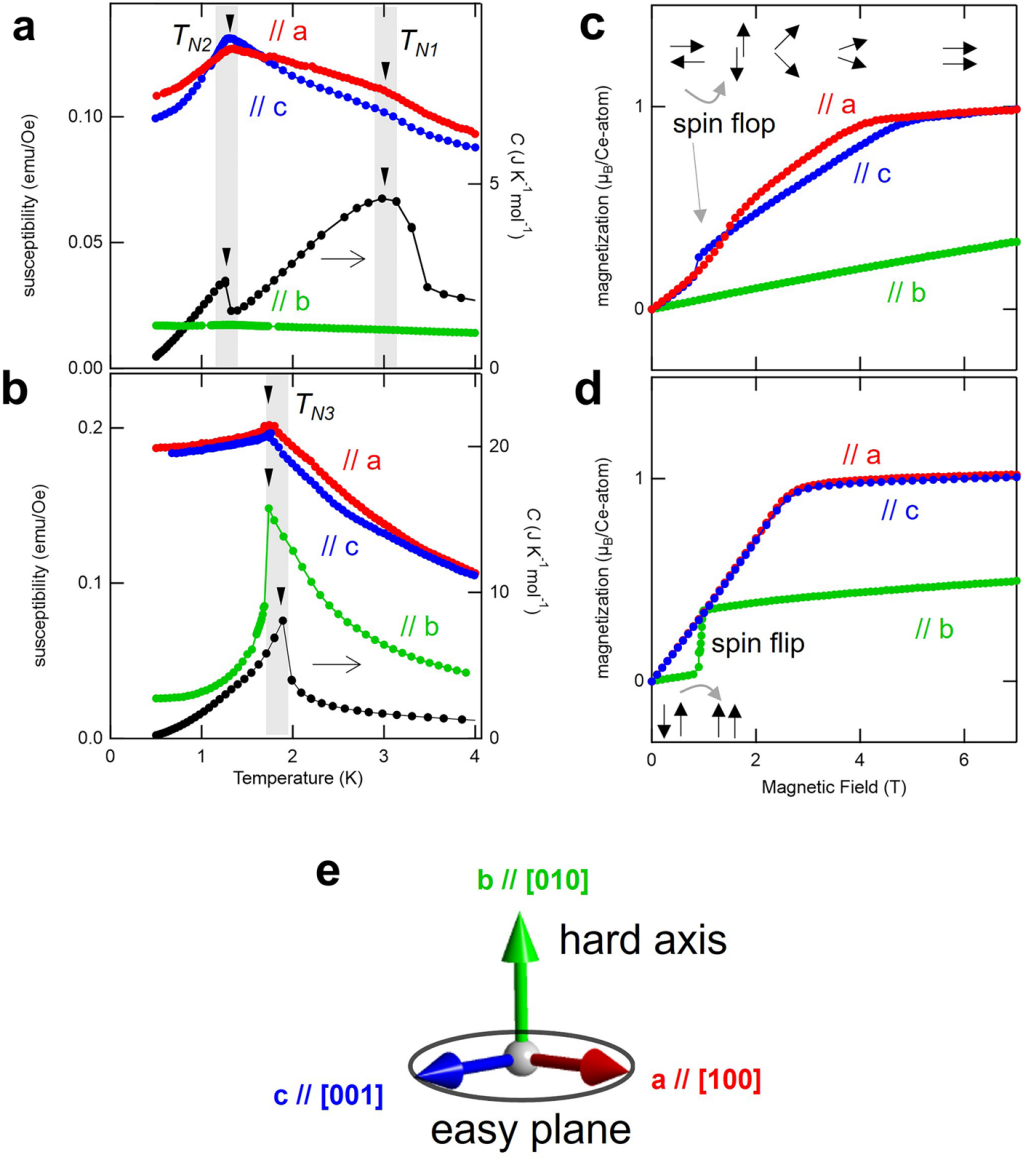
Magnetism and heat capacity characterization for CeTe_3_ and CeSeTe_2_ samples. (**a, b**) Temperature dependence of the magnetic susceptibility (right axis) for (**a**) CeTe_3_ and (**b**) CeSeTe_2_ with applying external field *H* = 0.1 T. On the right axis, temperature dependence of the heat capacity *C*(*T*) is also shown. In (**a**) and (**b**), there is slight difference in transition temperature since different samples were used for magnetic and heat capacity measurements. (**c**, **d**) Magnetic field dependence of the magnetization for (**c**) CeTe_3_ and (**d**) CeSeTe_2_ at *T* = 0.5 K. Cartoons for the magnetization process with spin-flop along easy plane and spin-flip along hard axis are shown in (**c**) and (**d**), respectively. The red, blue, and green data shown in (**a**–**d**) are obtained for *H* // a, *H* // c, and *H* // b, respectively. (**e**) The experimentally determined magnetic hard axis and easy plane together with crystal axes. These crystallographic and magnetic directions are the same for all samples shown in this study.

**Figure 3:**
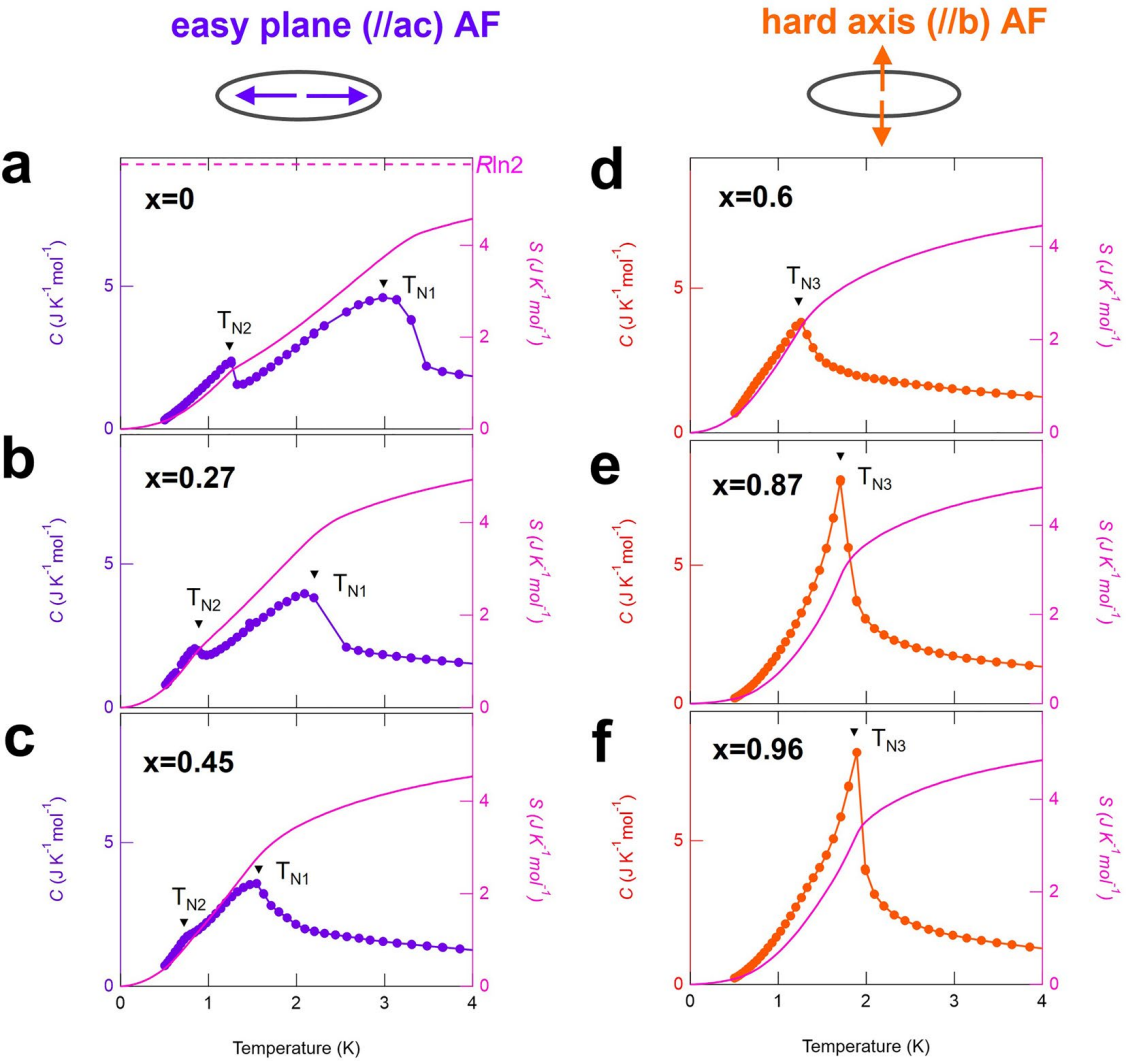
Doping and temperature dependence of heat capacity *C*(*T*) of Ce(Se_*x*_Te_1−*x*_)Te_2_. (**a**–**f**) Temperature dependence of the heat capacity (circles; left axis) and magnetic entropy (solid line; right axis) of (**a**) *x* = 0, (**b**) *x* = 0.27, (**c**) *x* = 0.45, (**d**) *x* = 0.60, (**e**) *x* = 0.87, (**f**) *x* = 0.96. The purple color represents data obtained from samples with easy plane antiferromagnetism (AF), and the orange represents those with hard axis AF. In (**a**), the value *R*ln2 (~ 5.76 JK^−1^ mol^−1^) is shown with a broken line. This value is the calculated magnetic entropy from the ground state doublet of Ce^3+^ ions under the crystalline electric field.

**Figure 4:**
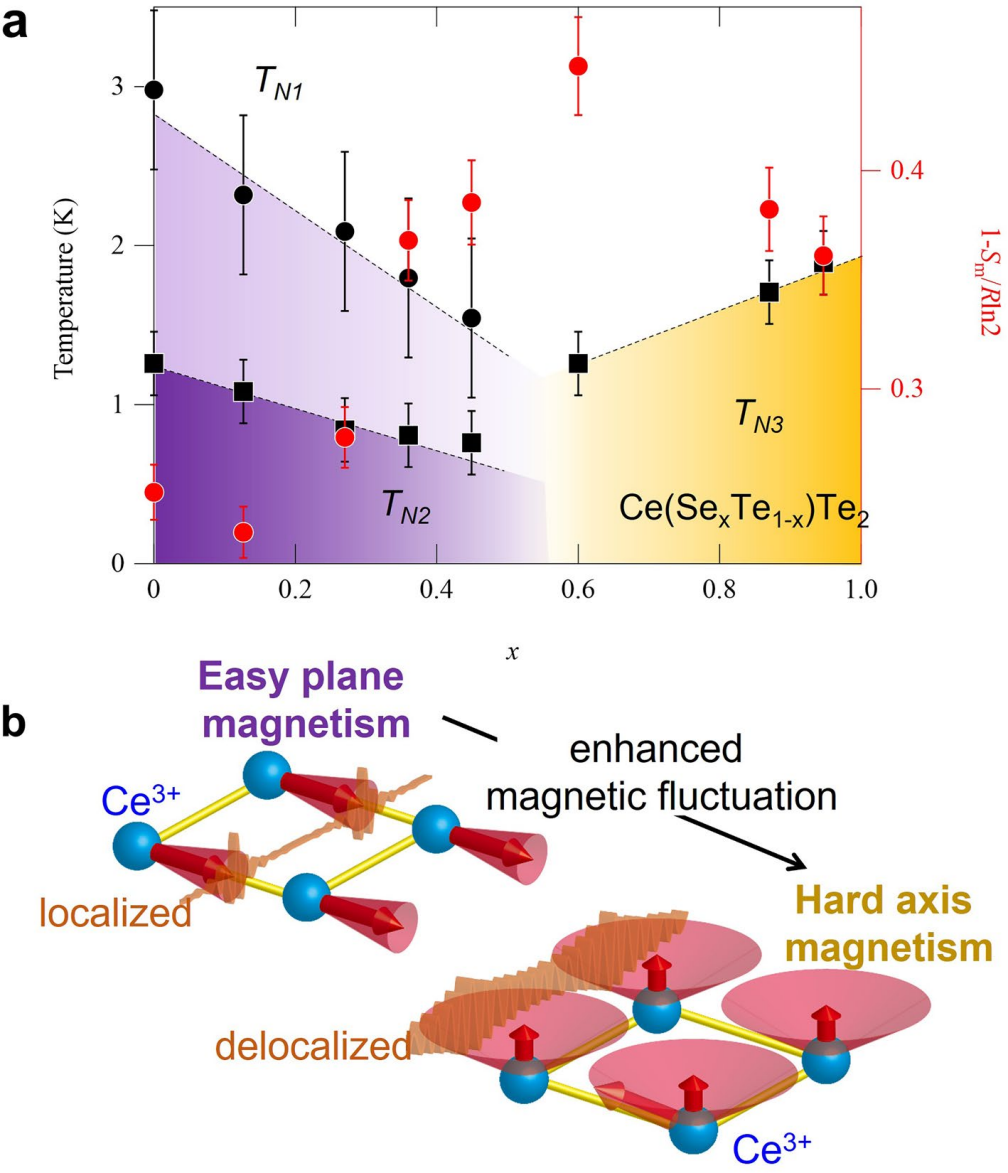
The phase diagram representing magnetic rotation associated with enhanced quantum fluctuation. (**a**) Temperature-doping phase diagram of Ce(Se_*x*_Te_1−*x*_)Te_2_ for three successive magnetic transition temperatures (*T*_N1_, *T*_N2_, and *T*_N3_) and magnetic entropies 1 − *S*_m_/*R*ln2. The data used in this phase diagram are from specific heat measurements. (**b**) Schematic drawing of Fermionic order by disorder. The magnetic moment lies in the easy axis (plane) and is reduced with enhanced quantum fluctuation for *x* < 0.54. Whereas with enhanced fluctuation the magnetic moment moves to lie along the hard axis (*x* > 0.54) and expresses enhanced precession as for an Ising-like moment. The kinetic energy gain with enhanced magnetic fluctuation is represented as a change from localized wave packet (left) to delocalized wave packet (right). As a detailed spin structure for antiferromagnetism within this compound is not totally clear, ferromagnetically aligned spins within single Ce square lattice sheet along ac plane (see Figs. 1a, 2a) are drawn for clarity.

