# Supplementary material for: Fermionic order by disorder in a van der Waals antiferromagnet
Source: Sci Rep. 2020 Sep 17;10:15311. doi: 10.1038/s41598-020-72300-3 (PMC7499234; doi:10.1038/s41598-020-72300-3)
Supplement: Supplementary file 1 — Supplementary information. [file 41598_2020_72300_MOESM1_ESM.docx]

**Fermionic Order by Disorder in a van der Waals Antiferromagnet**

R. Okuma^1*^, D. Ueta^1*^, S. Kuniyoshi^1,2*^, Y. Fujisawa^1^, B. Smith^1^, C. H. Hsu^1,3^, Y. Inagaki^4^,

W. Si^4^, T. Kawae^4^, H. Lin^5^, F. C. Chuang^3,6^, T. Masuda^7^, R. Kobayashi^2^, Y. Okada^1^

mail: [yoshinori.okada@oist.jp](mailto:yoshinori.okada@oist.jp)

*^1^Quantum Materials Science Unit, Okinawa Institute of Science and Technology (OIST),*

*Okinawa 904-0495, Japan*

*^2^Faculty of Science, University of the Ryukyus, Nishihara, Okinawa 903-0213, Japan*

*^3^Department of Physics, National Sun Yat-sen University, Kaohsiung 80424, Taiwan*

*^4^Department of Applied Quantum Physics, Kyushu University, Fukuoka 819-0395, Japan*

*^5^Institute of Physics, Academia Sinica, Taipei, Taiwan*

*^6^Physics Division, the National Center for Theoretical Sciences, Hsinchu, 30013, Taiwan*

*^7^Institute for Solid State Physics (ISSP), The University of Tokyo, Kashiwa, Chiba 277-8581, Japan*

**Pictures of samples**

| 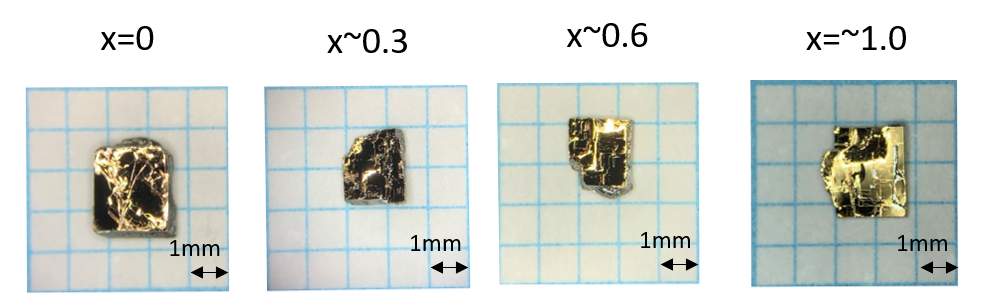 |
| --- |
| **Fig. S1**  The photos of Ce(Se_1-x_Te_x_)Te_2_ single crystals with various doping x. |

We succeeded in growing relatively large single crystals Ce(Se_1-x_Te_x_)Te_2_ with a plate-like morphology. All crystals are easy to exfoliate regardless of doping level. The systematic change in color with doping is consistent with systematic doping x.

**Phonon contribution**

| 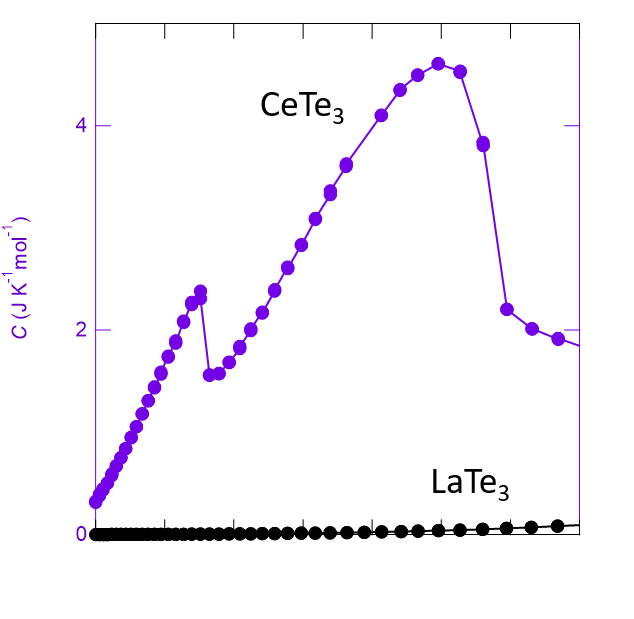 |
| --- |
| **Fig. S2**  Temperature dependence of the heat capacity for CeTe_3_ and LaTe_3_. |

The specific heat of LaTe_3_ is negligibly small compared to that of Ce(Se_1-x_Te_x_)Te_2_. This means 4f　electron derived specific heat contribution is dominant in Ce(Se_1-x_Te_x_)Te_2_. Thus, phonon subtraction is minor correction within the main purpose of this study.

**Band calculation**

The first-principles calculations were performed within the density functional theory (DFT) [1] using generalized gradient approximation of the Perdew-Burke-Ernzerhof (PBE) [2] and projector-augmented-wave (PAW) potentials [3], as implemented in the Vienna ab-initio simulation package (VASP) [4]. The kinetic energy cutoff was set at 500 eV. All atoms were relaxed using conjugate gradient method until the residual forces on each atom were smaller than 0.001 eV/$Å$. The Brillouin-zones (BZ) were sampled using $\Gamma$-centered 12$\times$2$\times$12 Monkhorst-Pack grid [5]. The optimized lattice parameters are a=c=4.422$Å$ and b=26.614$Å$ in theoretical calculation. SOC was included for all the band structure calculations. In order to account for the strong on-site Coulomb repulsion amongst the localized Ce 4f electrons, the DFT+U formalism formulated by Dudarev et al.[6] was used. $U_{eff}$ was set to 5 eV for the Ce 4f states, according to the previous literature [7-9]. Fig. S3 shows the band structure of CeTe_3_. The steep slope Dirac dispersion originates from *p* orbital of square net Te layers, with its Dirac point around -1.5eV. Another important feature is existence of *f*-orbital originated state near E_F_. Coexistence of *p*-orbital derived steep slope band and *f*-orbital derived flat band is a general important feature in this material.

| 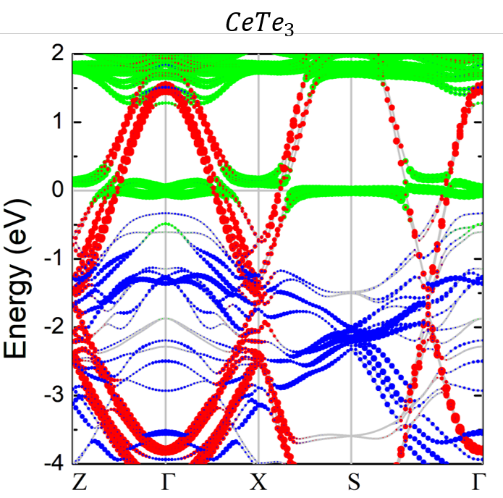 |
| --- |
| **Fig. S3**  Band structure for non-magnetic CeTe_3_. The red and blue circles represent the in-plane ($p_{x}$, $p_{z}$) and out-of-plane ($p_{y}$) orbitals which are contributions from the Te-plane, respectively. The green circles denote the Ce-4f orbitals. |

**Site selective Se doping**

To support site selective doping, we calculated free energies based on DFT. For a conventional CeTe_3_, there are four Ce atoms and twelve Te atoms in the unit cell. Thus, the doping concentration of one Se doping is 8.3%. Based on the structural symmetry, there are three sites which could be chosen for one Se doping (Fig. S4). In order to consider the stability, the relative energy ($E_{RE}$) was calculated by the formula, $E_{RE}=E_{\mathrm{doping}}-E_{\mathrm{Ce}{Te}_{3}}-N_{se}\times\mu_{Se}+N_{Te}\times\mu_{Te}$, where $E_{\mathrm{doping}}$ and $E_{\mathrm{Ce}{Te}_{3}}$ are the energy of doped and pure CeTe_3_, respectively. $\mu_{Se}$ and $\mu_{Te}$ are the chemical potentials of Se and Te bulk, respectively. $N_{se}$ and $N_{Te}$ are the number of Se dopants and Te replacements, respectively. We found that the Se doping within blocking later is the most probable case.

| 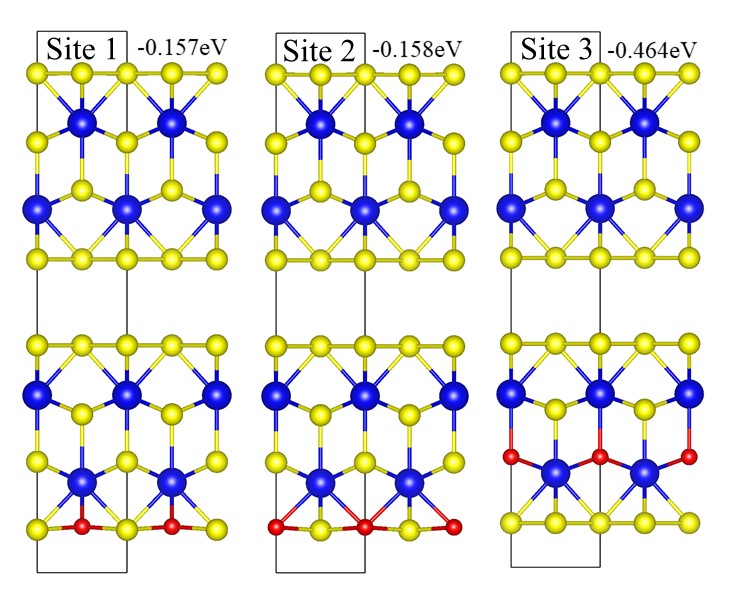 |
| --- |
| **Fig. S4**  The structural models for one Se dopant case. The atoms Te (yellow), Ce (blue), and Se (red) are shown with different color. The relative energies ($E_{RE}$) are also shown for three different cases. |

**References**

1. P. Hohenberg and W. Kohn, Phys. Rev. 136, B864 (1964). W. Kohn, and L. J. Sham, Phys. Rev. 140, A1133 (1965).
2. J. P. Perdew, K. Burke, and M. Ernzerhof, Phys. Rev. Lett. 77, 3865 (1996).
3. G. Kresse and D. Joubert, Phys. Rev. B 59, 1758 (1999).
4. G. Kresse and J. Hafner, Phys. Rev. B 47, 558 (1993). G. Kresse, and J. Furthmüller, Phys. Rev. B 54, 11169 (1996).
5. H. J. Monkhorst, and J. D. Pack, Phys. Rev. B 13, 5188 (1976).
6. S. L. Dudarev, G. A. Botton, S. Y. Savrasov, C. J. Humphreys, and A. P. Sutton, Phys. Rev. B 57, 1505 (1998).
7. C. W. M. Castleton, J. Kullgren, and K. Hermansson, J. Chem. Phys. 127, 244704 (2007)
8. M. Nakayama, H. Ohshima, M. Nogamia and M. Martin, Phys. Chem. Chem. Phys., 14, 6079–6084 (2012)
9. S. Grieshammer, Phys. Chem. Chem. Phys., 20, 19792 (2018)
